# Unveiling Silent Atherosclerosis in Type 1 Diabetes: The Role of Glycoprotein and Lipoprotein Lipidomics, and Cardiac Autonomic Neuropathy

**DOI:** 10.3390/metabo15010055

**Published:** 2025-01-16

**Authors:** Sara de Lope Quiñones, Manuel Luque-Ramírez, Antonio Carlos Michael Fernández, Alejandra Quintero Tobar, Jhonatan Quiñones-Silva, María Ángeles Martínez García, María Insenser Nieto, Beatriz Dorado Avendaño, Héctor F. Escobar-Morreale, Lía Nattero-Chávez

**Affiliations:** 1 Diabetes, Obesity and Human Reproduction Research Group, Instituto Ramón y Cajal de Investigación Sanitaria (IRYCIS) & Centro de Investigación Biomédica en Red Diabetes y Enfermedades Metabólicas Asociadas (CIBERDEM), Universidad de Alcalá, 28034 Madrid, Spain; 2 Department of Endocrinology and Nutrition, Hospital Universitario Ramón y Cajal, 28034 Madrid, Spain; jhonatanboris.quinones@salud.madrid.org (J.Q.-S.);; 3 Department of Radiology, Hospital Universitario Ramón y Cajal, 28034 Madrid, Spain

**Keywords:** cardioautonomic neuropathy, cardiac autonomic neuropathy, carotid plaques, glycoprotein profile, lipid profile, lipoproteins, metabolomics, proton nuclear magnetic resonance spectroscopy, subclinical atherosclerosis, triglycerides, type 1 diabetes mellitus

## Abstract

**Introduction:** This study aimed to evaluate whether glycoprotein and lipoprotein lipidomics profiles could enhance a clinical predictive model for carotid subclinical atherosclerosis in patients with type 1 diabetes (T1D). Additionally, we assessed the influence of cardiac autonomic neuropathy (CAN) on these predictive models. **Methods:** We conducted a cross-sectional study including 256 patients with T1D. Serum glycoprotein and lipoprotein lipidomics profiles were determined using ^1^H-NMR spectroscopy. Subclinical atherosclerosis was defined as carotid intima-media thickness (cIMT) ≥ 1.5 mm. CAN was identified using the Clarke score. Predictive models were built and their performance evaluated using receiver operating characteristic curves and cross-validation. **Results:** Subclinical atherosclerosis was detected in 32% of participants. Patients with both CAN and atherosclerosis were older, had a longer duration of diabetes, and were more likely to present with bilateral carotid disease. Clinical predictors such as age, duration of diabetes, and smoking status remained the strongest determinants of subclinical atherosclerosis [AUC = 0.88 (95%CI: 0.84–0.93)]. While glycoprotein and lipoprotein lipidomics profiles were associated with atherosclerosis, their inclusion in the clinical model did not significantly improve its diagnostic performance. Stratification by the presence of CAN revealed no impact on the model’s ability to predict subclinical atherosclerosis, underscoring its robustness across different risk subgroups. **Conclusions:** In a cohort of patients with T1D, subclinical atherosclerosis was strongly associated with traditional clinical risk factors. Advanced glycoprotein and lipoprotein lipidomics profiling, although associated with atherosclerosis, did not enhance the diagnostic accuracy of predictive models beyond clinical variables. The predictive model remained effective even in the presence of CAN, highlighting its reliability as a screening tool for identifying patients at risk of subclinical atherosclerosis.

## 1. Introduction

Cardiovascular disease (CVD) remains the leading cause of mortality among individuals with type 1 diabetes (T1D) [1]. This population faces an elevated risk of various cardiac complications, including accelerated atherosclerosis, cardiac autonomic neuropathy (CAN), and potentially intrinsic cardiomyopathy [2,3]. Among these, CAN has been closely linked to CVD in T1D [4]. The autonomic nervous system, which regulates heart rate and vascular tone, plays a critical role in maintaining cardiovascular health. Impaired autonomic function, as evidenced by reduced heart rate variability (HRV), may contribute to the development of subclinical atherosclerosis in patients with diabetes mellitus [5]. Preliminary findings from our research group [6] revealed a high prevalence of CAN in individuals with increased arterial stiffness and subclinical atherosclerosis. Our results demonstrated a significant association between reduced autonomic modulation and markers of arterial stiffness and atherosclerosis, suggesting that CAN may play a pivotal role in the early stages of vascular dysfunction [6].

Metabolomics and lipidomics, rapidly evolving fields over the past two decades, have become indispensable tools in biomedical research [7]. Among the techniques available, proton nuclear magnetic resonance (^1^H-NMRS) stands out as a robust and reliable method for simultaneously analyzing a broad spectrum of circulating metabolites with high precision and efficiency [8]. In addition to hyperglycemia, mounting evidence highlights the significant contribution of dyslipidemia to the development of atherosclerosis [9]. In this context, a ^1^H-NMRS-derived lipoprotein lipidomics panel provides detailed information on the composition, mean size, and concentration of diverse subtypes of lipoprotein particles, including large, medium, and small particles of the primary lipoprotein types—very low-density lipoprotein (VLDL), low-density lipoprotein (LDL), and high-density lipoprotein (HDL) [4].

In T1D, lipidomics studies, including several conducted by our research group, have identified alterations linked to microvascular complications such as CAN [4,10,11,12] and subclinical atherosclerosis [13]. Based on our previous findings regarding lipidomics and metabolomics biomarkers [4], we hypothesized that serum inflammatory glycoprotein and lipoprotein profiles could serve as early markers of atherosclerosis in individuals with T1D. Therefore, the objective of our study was to evaluate whether advanced serum glycoprotein and lipoprotein characteristics measured by ^1^H-NMRS can enhance a clinical predictive model for carotid subclinical atherosclerosis in patients with T1D. Additionally, we aimed to stratify participants based on the presence of CAN, to assess its potential influence on these predictive models.

## 2. Material and Methods

### 2.1. Study Population

**Figure 1:**
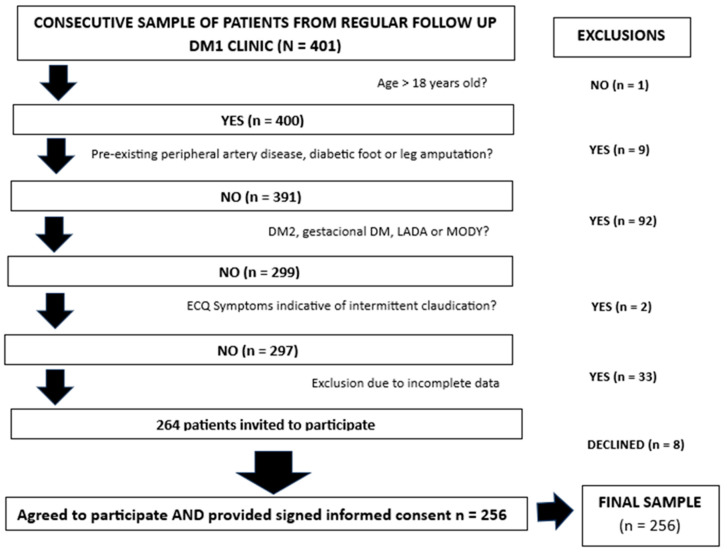
Flow chart of the study.

This cross-sectional study included 256 adult patients with T1D who were part of a larger cohort [14] regularly attending the diabetes outpatient clinic at an Academic Hospital in Madrid, Spain (ClinicalTrials.gov Identifier: NCT04950634) (Figure 1). Data collection was conducted between 2018 and 2021. Patients eligible for inclusion were required to meet the American Diabetes Association criteria for T1D, including a history of diabetic ketoacidosis and/or evidence of autoimmune diabetes, as well as insulin dependency for survival [15].

Exclusion criteria included the following: (i) age ≥ 85 years, (ii) inability to complete or comprehend the CAN assessment, (iii) presence of diabetic foot, (iv) end-stage renal disease or ongoing renal replacement therapy, (v) current pregnancy, and (vi) diagnosis of any type of diabetes mellitus other than T1D.

All participants provided written informed consent before inclusion in the study. No financial compensation was offered for participation.

### 2.2. Clinical and Laboratory Assessments

Medical history, current medications, and clinical parameters related to T1D were reviewed for all participants. A comprehensive anthropometric evaluation was performed, including measurements of weight, height, waist circumference, and hip circumference. The presence of microvascular complications—including diabetic retinopathy, neuropathy (defined as any neurological complication associated with T1D), and nephropathy (defined as any kidney disease related to T1D), as well as macrovascular complications (cerebrovascular disease, coronary artery disease, and peripheral arterial disease)—were documented [16]. The presence of diabetes complications was assessed through a comprehensive review of the patients’ medical history, physical examination (including foot examination), and relevant complementary tests. Microalbuminuria was evaluated using a first morning spot urine sample, collected on the same day as the cardiac autonomic assessment. Additionally, the most recent ophthalmological examination was reviewed to confirm or rule out the presence of diabetic retinopathy.

Fasting blood and urine samples were collected to assess renal function and measure the urinary albumin-to-creatinine ratio and glycated hemoglobin (A_1c_). Blood samples were immediately centrifuged, and serum and plasma aliquots were separated, coded, and stored at −80 °C for subsequent metabolomics and glycoprotein profile analyses.

### 2.3. Proton Nuclear Magnetic Resonance Spectroscopy Metabolomics

Frozen serum samples were transported on dry ice to Biosfer Teslab for lipoprotein lipidomics profile and glycoprotein quantification using ^1^H-NMR spectroscopy. Prior to analysis, each serum sample (200 µL) was mixed with 50 µL of deuterated water and 300 µL of a 50 mM phosphate buffer solution (PBS) at pH 7.4, prepared with 30.70 mM Na_2_HPO_4_ and 19.30 mM NaH_2_PO_4_.

The glycoprotein profile was determined using the Glycoscale^®^ assay (Biosfer Teslab, Tarragona, Spain), a methodology based on ^1^H-NMRS [17]. Spectra were recorded at 310 K on a Bruker Avance III 600 spectrometer, (Bruker Biospin, Rheinstetten, Germany) operating at 600.20 MHz (14.1 T). The profiling focused on the region between 2.15 and 1.90 ppm of the chemical shift. The line shape method was applied to distinguish the peaks corresponding to glycoproteins (GlycA and GlycB), which were further analyzed to calculate their areas and shape factors (height-to-width [H/W] ratios). The intra- and interassay variability of the ^1^H-NMRS method was less than 5% for all glycoproteins measured.

Lipid composition—including cholesterol (C) and triglycerides (TG)—as well as the mean size and particle number for the three primary lipoprotein types (VLDL, LDL, and HDL), were determined using the Liposcale^®^ assay (Biosfer Teslab, Tarragona, Spain). This approach categorized nine subtypes of lipoproteins: large, medium, and small VLDL, LDL, and HDL. The composition of intermediate-density lipoproteins (IDL) was also assessed [18].

### 2.4. Carotid Ultrasound Examination

The examination was conducted using the EPIQ Elite 5G ultrasound system (Philips, Amsterdam, Netherlands) equipped with 5 MHz to 12 MHz probes. Patients were examined in the supine position, and the carotid intima-media thickness (cIMT) was measured in both common carotid arteries. The mean of these two measurements was used for statistical analysis. Common carotid, internal carotid, external carotid, and vertebral arteries were also scanned for the presence of carotid plaques (CP). CP were defined as cIMT ≥ 1.5 mm protruding into the lumen. Subclinical carotid atherosclerosis was defined as the presence of at least one plaque in any of the territories explored. The criteria for categorizing arterial stenosis were defined as absent, mild (<50%), moderate (50–70%), severe (>70%); subtotal or total occlusion [19].

Participants rested in the supine position for a minimum of 10 min prior to blood pressure (BP) measurements. All vascular assessments were performed under standardized conditions (a quiet room with a comfortable temperature) following overnight fasting, to eliminate potential interference from postprandial glucose surges.

### 2.5. Assessment of Cardiovascular Autonomic Function: Ewing’s Score and Power Spectral Heart Rate Data

Cardiovascular autonomic function was evaluated using the tests described by Ewing et al. [20], as recommended by the American Diabetes Association’s consensus statement on standardized assessments for individuals with diabetes [21]. CAN was diagnosed based on two validated methods: (i) the standardized cardiac autonomic reflex tests (CARTs) developed by Ewing et al. in 1970 [20] and (ii) HRV analysis using power spectral analysis of beat-to-beat intervals from short-duration electrocardiogram (ECG) recordings. A detailed description of the methodology is available in our previous studies [8,14].

A modified Ewing score was used to diagnose CAN, incorporating the responses of HRV to deep breathing (E/I ratio), standing (30:15 ratio), Valsalva’s maneuver (VAL ratio), and the change in systolic blood pressure (∆SBP) during active standing. Responses were categorized as normal (0 points), borderline (0.5 points), or abnormal (1 point). A composite score ≥ 1 was diagnostic of CAN, which was further classified as early or mild (Ewing’s score 1–2) or definite (score ≥ 2).

HRV was measured using the VitalScan Medeia^®^ System (Medeia Inc., Santa Barbara, CA, USA). Participants were required to fast, excluding basal insulin, and abstain from food, nicotine, caffeine, and specific medications for 12 h before testing. Serum glucose levels were checked prior to testing to exclude hypoglycemia, and no participant had a glucose level < 70 mg/dL.

Adrenergic innervation was assessed by measuring BP and heart rate (HR) changes five minutes after standing. A BP difference of ≤10 mmHg was considered normal, 11–29 mmHg borderline, and ≥30 mmHg abnormal. Orthostatic hypotension was defined as a systolic BP drop > 20 mmHg. Resting HR was recorded by palpating the radial pulse, with HR > 100 beats per minute considered tachycardia.

Power spectral HRV data were obtained from 10 min ECG recordings analyzed using VitalScan Medeia^®^ software HW10. The Fourier transformation method was applied to R–R intervals, generating wavelets to identify low-frequency (LF, 0.04–0.15 Hz; sympathetic and parasympathetic influence) and high-frequency (HF, 0.15–0.4 Hz; parasympathetic activity) components.

## 3. Statistical Analysis

Descriptive statistics were used to summarize the data. Categorical variables were presented as frequencies and percentages, while numerical variables with a normal distribution (assessed using the Kolmogorov–Smirnov test) were expressed as means with standard deviations. Skewed numerical variables were log-transformed to evaluate normality, and if normality was not achieved, medians and interquartile ranges (IQR) were reported.

Differences in clinical and biochemical characteristics between the presence of CAN and preclinical atherosclerosis were analyzed using the χ^2^ test for categorical variables. For continuous variables, either the Mann–Whitney *U* test or Student’s *t*-test was applied as appropriate. Predictive factors of subclinical atherosclerosis among clinical features were identified by a binary logistic regression analysis. The following variables were included in the logistic regression model: sex (coded as 0 = women and 1 = men), age (years), duration of T1D (years), presence of CAN (coded as 0 = absent and 1 = present), dyslipidemia (coded as 0 = absent and 1 = present), hypertension (coded as 0 = absent and 1 = present), A_1c_ levels, insulin dose (IU/kg/day), smoking status (coded as 0 = absent and 1 = present), body fat (%), and estimated glomerular filtration rate (eGFR). These variables were selected based on their clinical relevance and potential association with subclinical atherosclerosis, as supported by the prior literature and the results of the univariate analysis.

The association between the lipoprotein lipidomics and glycoprotein profiles and preclinical atherosclerosis was assessed individually using binary logistic regression, adjusted for the most significant clinical variables identified after the initial adjustment: smoking status (coded as 0 = absent and 1 = present), duration of T1D (years), and age (years).

Performance metrics were assessed using receiver operating characteristic (ROC) curves. The diagnostic performance of the models based on isolated clinical factors, ^1^H-NMRS biomarkers, and the combination of clinical variables with ^1^H-NMRS biomarkers was assessed by ROC analyses and their areas under the curve (AUC). We compared AUC and ROC curves using the DeLong test. Additionally, a 5-fold cross-validation was conducted to assess the models’ predictive performance on unseen data, ensuring their generalizability.

Subjects with incomplete data were excluded only from the final analysis and model comparison to maintain the validity of results. Statistical significance was defined as a two-sided *p*-value < 0.05 for all analyses. Statistical computations were performed using the R statistical software environment [R Core Team (2024). _R: A Language and Environment for Statistical Computing. R Foundation for Statistical Computing, Vienna, Austria. https://www.R-project.org/ (accessed on 10 October 2024).

## 4. Results

### 4.1. Baseline Characteristics of the Study Population

A total of 256 participants with T1D were included, with a median age of 47 years (IQR 35–56) and a median diabetes duration of 24 years (IQR 16–34). Males accounted for 56% of the cohort. The median body mass index (BMI) was 25 (5) kg/m^2^, and the mean fat mass was 24 ± 10%. The median daily insulin dose was 0.5 IU/kg of body weight. Regarding treatment, 52% were on lipid-lowering therapy, 20% were receiving antihypertensive medication, and 11% were using antiplatelet therapy. Additionally, 32% (83 participants) reported current or prior smoking habits. These baseline characteristics are summarized in Table 1 and Table 2.

CAN was identified in 29% (75 participants) of the cohort. Participants with CAN were older, had a longer duration of diabetes, and showed a higher prevalence of hypertension, dyslipidemia, microangiopathy, and macroangiopathy compared to those without CAN (Table 1). Glycemic control was also poorer in participants with CAN, as evidenced by higher A_1c_ levels. Anthropometric differences included a significantly higher percentage of fat mass in participants with CAN (26 ± 8% vs. 24 ± 10%, *p* = 0.031), although BMI and waist-to-hip ratio did not differ significantly. These comparisons are detailed in Table 1.

Furthermore, patients with CAN were significantly more likely to present with atherosclerosis compared to those with normal cardioautonomic function [49% (36 out of 74) vs. 25% (45 out of 181), χ^2^ = 13.712, *p* < 0.001].

### 4.2. Clinical Characteristics Stratified by Subclinical Atherosclerosis Status

Subclinical atherosclerosis was identified in 32% of participants (81 individuals), with bilateral involvement observed in 48% (39 cases) of those affected. Similarly to patients with CAN, participants with subclinical atherosclerosis were older (median age: 58 (13) vs. 42 (19) years, *p* < 0.001) and had a longer duration of diabetes (median: 32 (18) vs. 21 (17) years, *p* < 0.001). As expected, smoking habits were significantly more prevalent among participants with atherosclerosis. The presence of subclinical atherosclerosis was also associated with a higher prevalence of hypertension, microangiopathy, and macroangiopathy. Additionally, participants with subclinical atherosclerosis had a lower estimated glomerular filtration rate (eGFR) compared to those without (83 ± 14 vs. 92 ± 16 mL/min/1.73 m^2^, *p* < 0.001), and slightly higher A_1c_ levels (7.4 ± 0.9% vs. 7.1 ± 0.9%, *p* = 0.041). These findings are summarized in Table 2.

In the multivariable model, the age [OR: 1.13 (95%CI: 1.07–1.20), *p* < 0.001], duration of diabetes [OR: 1.04 (95%CI: 1.00–1.08), *p* = 0.030], and smoking status [(OR: 2.54 (95%CI: 1.15–5.75), *p* = 0.020] were the principal clinical predictors of subclinical atherosclerosis. In contrast, the presence of CAN, dyslipidemia, hypertension, fat mass percentage, A_1c_ levels, and eGFR were not statistically significant, despite showing significant associations in univariate analyses (Table 3).

When comparing participants with both atherosclerosis and CAN to those with atherosclerosis but normal cardioautonomic function, distinct clinical characteristics were observed. Individuals with both conditions were significantly older (62 ± 9 vs. 55 ± 8 years, *p* < 0.001), had a longer duration of diabetes (36 ± 10 vs. 29 ± 11 years, *p* = 0.004), and displayed higher SBP levels (133 ± 15 vs. 125 ± 17 mmHg, *p* = 0.027). Furthermore, patients with CAN were more likely to have bilateral atherosclerotic disease compared to those without CAN (56% vs. 43%, χ^2^ = 4.362, *p* = 0.037).

### 4.3. Lipid Profile and Glycoprotein Quantification Using ^1^H-NMR Spectroscopy

Patients with T1D and atherosclerotic plaques exhibited worse lipoprotein lipidomics and inflammatory profiles compared to those without atherosclerosis (Table 4). These individuals demonstrated elevated levels of lipoprotein-associated cholesterol and triglycerides, along with a higher concentration of small VLDL particles, a reduced VLDL diameter, and increased numbers of medium LDL and large HDL particles. Additionally, all glycoprotein biomarkers, as well as fibrinogen, were significantly elevated in patients with atherosclerosis (Table 4).

**Figure 2:**
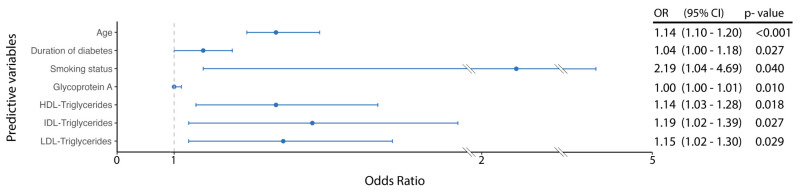
Multivariate model adjusted for clinical predictors of the presence of atherosclerosis (age, duration of diabetes, and smoking), as well as glycoproteins and lipid profiles (Glyc A, IDL-TG, LDL-TG, and HDL-TG).

In a multivariable model adjusted for clinical predictors (age, diabetes duration, and smoking status), only Glyc A [OR: 1.00 (95%CI: 1.00–1.01); *p* = 0.010], IDL-TG [OR: 1.19 (95%CI: 1.02–1.39); *p* = 0.027], LDL-TG [OR: 1.15 (95%CI: 1.02–1.30); *p* = 0.029], and HDL-TG [OR: 1.14 (95%CI: 1.03–1.28); *p* = 0.018] remained statistically significant. The impact of these metabolites on the adjusted model is shown in Figure 2.

### 4.4. Predictive Performance Analysis of the Model

**Figure 3:**
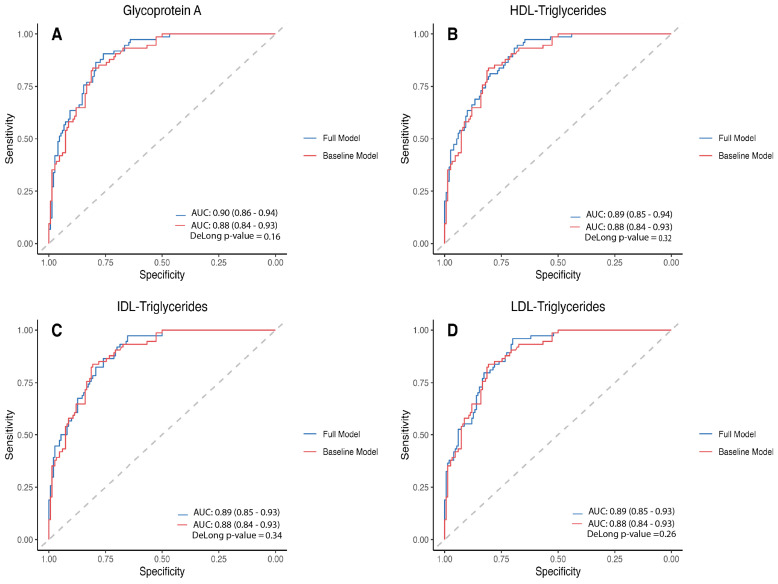
Receiver operating characteristic (ROC) curves comparing the area under the curve (AUC) of the clinical model, which includes age, duration of T1D, and smoking status. AUC of Glycoprotein A (**A**), HDL-Triglycerides (**B**), IDL-Triglycerides (**C**), and LDL-Triglycerides (**D**).

The baseline clinical model, incorporating age, duration of diabetes, and smoking status, demonstrated strong predictive performance with an AUC of 0.88 (95%CI: 0.84–0.93). Adding GlycA to the clinical model resulted in an AUC of 0.90 (95%CI: 0.86–0.94), not statistically significant (DeLong’s test, *p* = 0.16). Similar results were observed when other metabolites were added to the baseline model: (i) HDL-TG: AUC = 0.89 (95%CI: 0.85–0.93); DeLong’s test, *p* = 0.32; (ii) IDL-TG: AUC = 0.89 (95%CI: 0.85–0.93); DeLong’s test, *p* = 0.34; and (iii) LDL-TG: AUC = 0.89 (95%CI: 0.85–0.93); DeLong’s test, *p* = 0.26. When adding the four metabolites to the clinical prediction model, we obtained an AUC of 0.902 (95% CI: 0.863–0.940). When compared with the baseline model, the DeLong test yielded a *p*-value of 0.1. However, upon performing a 5-fold cross-validation, the performance of the model achieved an AUC of 0.88, which is similar to the AUC of the other models in the test set (Supplementary Figure S1). Therefore, despite the strong association of these four metabolites with subclinical atherosclerosis, their addition to the clinical model does not improve the model’s predictive power for subclinical atherosclerosis. Figure 3 shows the ROC curves, comparing the baseline clinical model with each of the models incorporating one of these metabolites. To assess the model’s performance on unseen data, a 5-fold cross-validation was conducted (Supplementary Figure S2). The baseline model achieved a mean AUC of 0.88 on the test sets, closely reflecting its performance on the training data with a good generalizability. Similarly, the cross-validated AUCs for the models incorporating GlycA, HDL-TG, IDL-TG, and LDL-TG were all 0.88, indicating consistent performance across both the training and test sets.

To enhance the baseline clinical model as a screening tool, the probability threshold was adjusted to 0.18. At this threshold, the model achieved a sensitivity of 93% (95%CI: 85–98) and a negative predictive value of 95% (95%CI: 89–98), ensuring high reliability in excluding subclinical atherosclerosis. However, this optimization was accompanied by a specificity of 63% (95%CI: 55–71) and a positive predictive value of 56% (95%CI: 46–65) (Supplementary Table S1). These results suggest that setting the probability threshold at 0.18 became the model well suited for screening purposes, effectively identifying patients at risk of subclinical atherosclerosis.

The model retained its validity even after stratifying participants based on the presence of CAN (Supplementary Figure S3), highlighting its robustness across different risk subgroups [AUC in patients with CAN = 0.88 (95%CI: 0.81–0.96); AUC in patients without CAN = 0.88 (95%CI: 0.83–0.93)]. When stratifying the analysis by age using a cutoff of 50 years, differences in the ROC curves were observed (Supplementary Figure S4). The ROC curve for patients under 50 years of age showed an AUC of 0.87 (95% CI: 0.80–0.94), while the ROC curve for patients aged 50 years or older had an AUC of 0.75 (95% CI: 0.64–0.86). Although the DeLong test yielded a *p*-value of 0.06, suggesting a trend toward a difference between the two curves, further analysis indicated that smoking history had a greater influence on the predictive model for patients under 50 years of age. These findings highlight the potential age-dependent impact of smoking on the model’s performance.

## 5. Discussion

Atherosclerosis is one of the most critical and severe cardiovascular complications in patients with T1D [22,23]. In routine clinical practice, atherosclerosis is often undetected among patients with T1D. Despite this, current clinical practice guidelines do not recommend routine screening for asymptomatic patients, underscoring a significant gap in preventive care. As a result, there is substantial interest in developing screening strategies to identify subclinical atherosclerosis in order to, this way, mitigate the risk of future cardiovascular events. In this study, we aimed to evaluate the presence of subclinical atherosclerosis with advanced lipoprotein lipidomics profiling and protein glycation analysis through ^1^H-NMR spectroscopy. Our findings revealed that one out of three asymptomatic patients with T1D had subclinical atherosclerosis, as detected by carotid ultrasound imaging, highlighting its high prevalence among individuals with T1D (similar to the reported prevalence of atherosclerosis in this population, approximately 5–35%) [22,24,25]. Logistic regression models in our cohort identified age, duration of T1D, and smoking exposure as the main clinical determinants associated with subclinical atherosclerosis. The relevance of our work lies in demonstrating that, with just three clinical variables, our model achieves excellent diagnostic performance in predicting subclinical atherosclerosis. These findings reinforce the clinical relevance of readily available variables in identifying patients at higher risk. The implementation of this model in routine practice could transform patient management by allowing a proactive and personalized approach to targeting diagnostic testing in high-risk patients to mitigate long-term cardiovascular complications. Our model retained its validity even after stratifying participants based on the presence of CAN, highlighting its robustness across different risk subgroups. Although ^1^H-NMR-derived lipoprotein and glycoprotein analysis revealed striking associations with atherosclerosis, it did not enhance the predictive power of our clinical model, which was already highly robust.

Recently, there has been growing interest in the role of triglyceride-rich lipoproteins in the pathogenesis of cardiovascular diseases. The relationship between glucose and lipid metabolism is well established, with hypertriglyceridemia and reduced HDL levels frequently co-existing in patients with poorly controlled T1D [26]. Advances in high-throughput lipidomics analysis offer the potential to uncover novel pathways and biomarkers, providing deeper insights into the mechanisms of cardiovascular disease in T1D and identifying new targets for prevention and intervention [27]. The relationships between lipoprotein and glycoprotein profiles assessed by ^1^ H-NMRS and atherogenicity have been reported in individuals with T1D [25,28]. A recent study demonstrated a direct association between the triglyceride content in LDL particles (LDL-TG) and the presence of atherosclerosis. Similarly, triglyceride-rich HDL particles, which exhibit atherogenic properties, appear to be more prevalent in patients with T1D [29]. Moreover, it has been shown that the abundance of these atherogenic HDL particles decreases with improved glycemic control [30], further emphasizing the intricate relationship between glucose and lipid metabolism.

Indeed, our cohort of patients exhibited good metabolic control, including glycemic and lipid parameters, with optimal TG levels. However, the association between TG-rich lipoprotein metabolites and atherosclerosis in T1D is a complex and well-documented phenomenon that goes beyond traditional lipid profiles [28,31]. Even in individuals with optimal lipid levels, T1D is often associated with subtle but clinically significant alterations in lipoprotein composition and function. These changes include the enrichment of triglycerides in intermediate-density lipoproteins (IDL-TG) and low-density lipoproteins (LDL-TG), which may increase their atherogenic potential [32]. Research has shown that TG-enriched lipoproteins are more prone to oxidation and can promote endothelial dysfunction and foam cell formation, which are key steps in atherogenesis. Several studies have linked triglyceride-rich lipoproteins (TRLs) to increased cardiovascular risk. TG-rich metabolites have been linked to enhanced immune responses and arterial inflammation, further contributing to atherosclerosis [31]. In individuals with T1D, even with good metabolic control, insulin deficiency or resistance at the vascular level may impair the normal metabolism of TG-rich particles. This can lead to the persistence of atherogenic TG-rich lipoproteins in circulation. Additionally, systemic low-grade inflammation and endothelial dysfunction commonly observed in T1D further amplify the effects of these metabolites on atherosclerotic processes. In summary, TG-rich metabolites are strongly associated with atherosclerosis in T1D due to their atherogenic properties, even in the absence of hypertriglyceridemia. This association highlights the importance of evaluating not only traditional lipid parameters but also advanced lipidomic profiles, to better understand cardiovascular risk in this population.

In the same way, inflammation is recognized as a key driver in the development and progression of atherosclerosis [33], with a well-established link between classical inflammatory markers and atherosclerotic cardiovascular events [34]. Patients with T1D consistently exhibit higher levels of classical inflammatory markers compared to individuals without diabetes [35,36,37]. Among these markers, GlycA has emerged as an important biomarker, associated with elevated mortality risk in patients with cardiovascular disease and atherogenic dyslipidemia [38]. Notably, circulating GlycA levels correlate with hs-CRP, a widely used inflammatory biomarker in clinical practice [38]. However, GlycA may offer additional specificity, suggesting that the glycoprotein profile could provide more precise information than hs-CRP in assessing inflammation [39].

Nonetheless, the incorporation of glycoprotein and lipoprotein profiles did not improve the diagnostic performance of the predictive model in our series. One possible explanation for this may be that these biomarkers are more closely related to cardiovascular risk factors than to the presence of atherosclerosis. Supporting this theory are findings from previous studies that reported a positive association between GlycA and cardiovascular risk factors such as smoking, BMI, hypertension, and dyslipidemia, suggesting that modifiable lifestyle risk factors influence the expression of glycoproteins producing the GlycA signal [40]. Similarly, relationships between plasma glycans and cardiovascular risk factors have been observed in studies employing techniques such as high-performance liquid chromatography [41]. Therefore, although modified glycoproteins and lipoproteins are associated with atherosclerosis, their strong correlation with cardiovascular risk factors may reduce their incremental value in enhancing predictive models for atherosclerosis.

In the present study, we also stratified patients with T1D based on the presence of CAN. Our findings demonstrate that patients with CAN are more likely to develop subclinical atherosclerosis. Furthermore, those with both CAN and subclinical atherosclerosis tend to be older and have a longer duration of diabetes. Notably, these patients also exhibit an increased probability of presenting with bilateral atherosclerosis. Given that CAN is another condition associated with increased cardiovascular mortality and potentially represents a distinct phenotypic presentation, it is critical to ensure that predictive models accurately diagnose atherosclerosis in the presence of CAN. Interestingly, the presence of CAN did not influence the diagnostic performance of the predictive model for carotid atherosclerosis, reinforcing the robustness of the model across this high-risk subgroup.

Our study has several strengths. The study sample consisted of a large number of patients with well-controlled T1D assessed by a detailed protocol with state-of-the-art methodology. All patients were subjected to ultrasound imaging of carotid arteries, irrespective of the presence or absence of atherosclerosis. We used a model adjusted for various possible confusion factors and multiplicity, stratified by the presence of CAN.

However, our study was not free of limitations either. The cross-sectional design of our study limits our ability to establish causality. Another limitation of our study is the lack of a formal sample size calculation, as the cohort was intentionally selected from a larger population of patients regularly attending our clinic. While this approach ensured the inclusion of a representative sample with sufficient clinical and metabolic data, it may have limited our ability to detect small but potentially significant differences in the metabolomic and lipidomic profiles. Future studies with larger, adequately powered cohorts are warranted to validate these findings and explore their broader applicability. Secondly, our study applied a targeted metabolomic approach, focusing on a specific subset of metabolites in serum. This limited scope may restrict our ability to comprehensively understand the broader metabolomics landscape, as the blood metabolome may not necessarily reflect tissue-specific abnormalities. Another limitation of our study is that subclinical atherosclerosis was only evaluated at the carotid level, which may not fully capture the behavior of atherosclerosis in other vascular territories. Future studies should aim to include a broader assessment of subclinical atherosclerosis in additional vascular locations to provide a more comprehensive understanding of the disease. Moreover, the majority of patients were Caucasian Spaniards. Considering that our rates of cardiovascular disease are among the lowest in the world, this lack of ethnic diversity may not be, in part, representative of other populations. We included a considerable proportion of young patients, which may have influenced our prevalence figures. Our cohort is a cohort with good glycemic control. On the other hand, the use of statins in some patients could have influenced our results, due to their effect of atheroprotection.

## 6. Conclusions

In summary, our study highlights the significant prevalence of subclinical atherosclerosis in asymptomatic patients with T1D, emphasizing the need for early detection strategies to mitigate the progression of cardiovascular disease in this high-risk population. While glycoprotein and lipoprotein lipidomics profiles were associated with cardiovascular risk factors, their incorporation into predictive models did not improve their diagnostic performance beyond that of classic clinical variables, underscoring the critical role of traditional risk factors such as age, duration of diabetes, and smoking status. Furthermore, stratification by the presence of CAN revealed that patients with CAN are not only more likely to develop atherosclerosis, but also exhibit a distinct phenotype characterized by older age, longer diabetes duration, and an increased likelihood of bilateral carotid disease. Importantly, the diagnostic accuracy of the predictive model for atherosclerosis remained robust even in the presence of CAN. These findings pave the way for future research into phenotypic variations and tailored interventions in this population.

## Figures and Tables

**Table 1:** Demographic and clinical characteristics of all study participants as a function of the presence or absence of CAN.

Variable	All Patients	Presence of CAN		*p*
	(n = 256)	Yes (n = 75)	No (n = 181)	
Male/female (%)	142 (56)/114 (44)	42 (56)/33 (44)	100 (55)/81 (45)	0.912
Age (yrs)	47 (21)	56 (16)	44 (18)	**<0.001**
Duration of T1D (yrs)	24 (18)	31 (18)	21 (17)	**<0.001**
Smoking habit [N (%)]	83 (32)	31 (41)	52 (29)	0.070
Daily insulin dose (units/kg/day)	0.52 (0.22)	0.54 (0.24)	0.51 (0.21)	0.964
Antiaggregant therapy [N (%)]	28 (11)	20 (27)	8 (4.4)	**<0.001**
Antihypertensive therapy [N (%)]	52 (20)	29 (39)	23 (13)	**<0.001**
Microangiopathy [N (%)]	81 (32)	40 (53)	41 (23)	**<0.001**
Macroangiopathy [N (%)]	12 (4.7)	7 (9.3)	5 (2.8)	**0.047**
Dyslipemia [N (%)]	134 (52)	53 (71)	81 (45)	**<0.001**
Body mass index (kg/m^2^)	25 (5.1)	26 (4.9)	25 (5.7)	0.061
Fat mass (%)	24 ± 9.5	26 ± 8	24 ± 10	**0.031**
Waist-to-hip ratio	0.93 (0.12)	0.94 (0.10)	0.93 (0.13)	0.139
Systolic BP (mmHg)	119 (19)	127 (19)	117 (15)	**<0.001**
Diastolic BP (mmHg)	74 (10)	76 (9)	74 (9)	**0.026**
eGFR (mL/min/1.73 m^2^)	89 ± 16	87 ± 17	90 ± 15	0.134
A_1c_ (%)	7.2 ± 0.9	7.5 ± 1.0	7.1 ± 0.8	**0.001**
Total cholesterol (mg/dL)	170 (42)	173 (59)	169 (34)	0.622
HDL-cholesterol (mg/dL)	59 ± 15	60 ± 17	59 ± 14	0.828
LDL-cholesterol (mg/dL)	99 ± 24	102 ± 29	98 ± 23	0.413
Triglycerides (mg/dL)	58 (26)	61 (26)	57 (27)	0.177
Microalbumin/creatinine ratio (mg/g)	5.8 (4.6)	5.6 (4.0)	6 (5.4)	0.623

Continuous variables are shown as mean ± SD or median (IQR). Discrete variables are shown as raw numbers (percentage). The differences between groups were analyzed by parametric (Student’s *t*-test) or non-parametric (Mann–Whitney U) tests. Abbreviations: eGFR, estimated glomerular filtration rate; A_1c_, glycated hemoglobin.

**Table 2:** Demographic and clinical characteristics of all study participants as a function of the presence or absence of carotid plaque.

Variable	All Patients	Presence of Carotid Plaque		*p*
	(n = 256)	Yes (n = 81)	No (n = 175)	
Male/female (%)	142 (56)/114 (44)	46 (57)/35 (43)	95 (55)/79 (45)	0.743
Age (yrs)	47 (21)	58 (13)	42 (19)	***<0.001***
Duration of T1D (yrs)	24 (18)	32 (18)	21 (17)	***<0.001***
Smoking habit [N (%)]	83 (32)	41 (51)	41 (24)	***<0.001***
Daily insulin dose (units/kg/day)	0.52 (0.22)	0.49 (0.27)	0.52 (0.21)	0.659
Antiaggregant therapy [N (%)]	28 (11)	21 (26)	6 (3)	***<0.001***
Hypertension [N (%)]	52 (20)	33 (41)	18 (10)	***<0.001***
Microangiopathy [N (%)]	81 (32)	44 (54)	37 (21)	***<0.001***
Macroangiopathy [N (%)]	12 (4.7)	9 (11)	2 (1)	***0.001***
Dyslipemia [N (%)]	134 (52)	68 (84)	65 (37)	***<0.001***
Body mass index (kg/m^2^)	25 (5.1)	26 (6.6)	25 (4.7)	***0.022***
Fat mass (%)	24 ± 9.5	26 ± 9.1	23 ± 9.7	***0.024***
Waist-to-hip ratio	0.93 (0.12)	0.95 (0.13)	0.93 (0.13)	***0.039***
Systolic BP(mmHg)	119 (19)	126 (23)	117 (16)	***<0.001***
Diastolic BP(mmHg)	74 (10)	76 (9)	73 (9)	***0.003***
eGFR (mL/min/1.73 m^2^)	89 ± 16	83 ± 14	92 ± 16	***<0.001***
A_1c_ (%)	7.2 ± 0.9	7.4 ± 0.9	7.1 ± 0.9	***0.041***
Total cholesterol (mg/dL)	170 (42)	177 (45)	168 (38)	0.159
HDL-cholesterol (mg/dL)	59 ± 15	60 ± 18	59 ± 13	0.601
LDL-cholesterol (mg/dL)	99 ± 24	101 ± 25	98 ± 24	0.491
Triglycerides (mg/dL)	58 (26)	64 (37)	56 (24)	***0.001***
Microalbumin/creatinine ratio (mg/g)	5.8 (4.6)	6.8 (6)	5.4 (3.9)	***0.031***

Continuous variables are shown as mean ± SD or median (IQR). Discrete variables are shown as raw numbers (percentage). The differences between groups were analyzed by parametric (Student’s *t*-test) or non-parametric (Mann–Whitney U) tests. Abbreviations: eGFR, estimated glomerular filtration rate; A_1c_, glycated hemoglobin.

**Table 3:** The multivariable model of baseline characteristics for the presence of subclinical atherosclerosis.

Variable	OR (95%IC)	*p*
Sex (male)	1.51 (0.51–4.66)	0.460
Age (years)	1.13 (1.07–1.20)	***<0.001***
Duration of T1D (years)	1.04 (1.00–1.08)	***0.028***
Daily insulin dose (units/kg/day)	0.75 (0.07–7.22)	0.804
Presence of CAN	0.72 (0.29–1.70)	0.455
Smoking habit	2.54 (1.15–5.75)	***0.022***
Hypertension	1.48 (0.59–3.69)	0.403
Dyslipemia	1.54 (0.48–5.30)	0.474
Fat mass (%)	0.98 (0.92–1.03)	0.399
A_1c_ (%)	1.06 (0.68–1.65)	0.792
eGFR (mL/min/1.73 m^2^)	0.98 (0.95–1.01)	0.243

Abbreviations: eGFR, estimated glomerular filtration rate; A_1c_, glycated hemoglobin.

**Table 4:** The univariate logistic regression model for the statistically significant metabolite determinants of subclinical atherosclerosis.

	All Patients (n = 256)	Presence of Atherosclerosis		
		Yes (n = 81)	No (n = 175)	*p*
**Cholesterol (C)**				
VLDL-C (mg/dL) *	10 ± 7	11 ± 8	9 ± 6	0.041
IDL-C (mg/dL) ***	9 ± 4	10 ± 4	8 ± 3	<0.001
LDL-C (mg/dL)***	117 ± 19	120 ± 20	116 ± 18	<0.001
HDL-C (mg/dL)	64 ± 12	65 ± 14	63 ± 11	0.202
**Triglycerides (TG)**				
VLDL-TG (mg/dL)	46 ± 29	51 ± 29	45 ± 29	0.074
IDL-TG (mg/dL) ***	10 ± 3	11 ± 3	9 ± 2	<0.001
LDL-TG (mg/dL) ***	13 ± 4	15 ± 4	12 ± 3	<0.001
HDL-TG (mg/dL) ***	14 ± 4	16 ± 5	13 ± 4	<0.001
**Lipoprotein particle number**				
VLDL-P (nM) *	33.5 ± 19.7	36.8 ± 20.4	31.9 ± 19.2	0.021
Large VLDL-P (nM)	0.9 ± 0.4	1.0 ± 0.4	0.9 ± 0.4	0.052
Medium VLDL-P (nM)	3.4 ± 3.0	3.6 ± 2.9	3.3 ± 3.1	0.623
Small VLDL-P (nM) **	29.2 ± 16.5	32.3 ± 17.3	27.7 ± 16.0	0.008
LDL-P (nM) *	1153 ± 179	1191 ± 203	1136 ± 165	0.030
Large LDL-P (nM)	184 ± 26	186 ± 27	183 ± 26	0.439
Medium LDL-P (nM) *	345 ± 93	365 ± 95	336 ± 90	0.021
Small LDL-P (nM)	625 ± 95	641 ± 111	617 ± 85	0.083
HDL-P (µM) *	31.0 ± 5.0	32.2 ± 5.4	30.5 ± 4.7	0.015
Large HDL-P (µM) ***	0.3 ± 0.0	0.3 ± 0.1	0.3 ± 0.0	<0.001
Medium HDL-P (µM) *	11.4 ± 2.3	11.9 ± 2.5	11.1 ± 2.2	0.020
Small HDL-P (µM)	19.3 ± 3.5	20.0 ± 3.7	19.1 ± 3.4	0.060
**Lipoprotein particle size**				
VLDL diameter (nm) **	42.2 ± 0.2	42.1 ± 0.2	42.2 ± 0.2	0.009
LDL diameter (nm)	21.1 ± 0.2	21.1 ± 0.2	21.1 ± 0.2	0.981
HDL diameter (nm)	8.3 ± 0.1	8.3 ± 0.1	8.3 ± 0.1	0.581
**Inflammation**				
Glyc A (µmol/L) **	607 ± 117	637 ± 125	593 ± 111	0.002
Glyc A H/W **	14.9 ± 2.4	15.6 ± 2.5	14.6 ± 2.2	0.001
Glyc B (µmol/L) **	316 ± 42	327 ± 43	311 ± 41	0.006
Glyc B H/W **	4.0 ± 0.5	4.1 ± 0.5	3.9 ± 0.5	0.005
Glyc F (µmol/L) *	202 ± 37	207 ± 35	199 ± 38	0.032
Fibrinogen (mg/dL) ***	299 ± 79	330 ± 90	284 ± 68	<0.001
hs-CRP (mg/L)	0.9 (1.89)	1.09 (1.80)	0.83 (1.84)	0.115

Data are expressed as mean ± SD or median (IQR). The differences between groups were analyzed by parametric (one-way analysis of variance) or non-parametric (Mann–Whitney U) tests. Abbreviations: Glyc A, N-acetylglucosamine/galactosamine; Glyc B, sialic acid; HDL, high-density lipoprotein; hs-CRP, high-sensitive C reactive protein; H/W, height to width ratio; IDL, intermediate-density lipoprotein; LDL, low-density lipoprotein; and VLDL, very low density lipoprotein.

## Data Availability

The data that support the findings of this study are available from the corresponding author [LN-C] upon reasonable request. The data are not publicly available because they contain information that could compromise the privacy of research participants.

## Supplementary Materials

The following supporting information can be downloaded at: https://www.mdpi.com/article/10.3390/metabo15010055/s1, Figure S1: ROC curves comparing the baseline clinical prediction model and the model incorporating the four significant biochemical metabolites; Figure S2: ROC curves representing the validation of the predictive models; Table S1: Confusion matrix for the prediction model at a cut-off point of 0.18; Figure S3: ROC curves comparing the predictive performance of the model adjusted for the presence of cardiac autonomic neuropathy (CAN); Figure S4: ROC curves stratified by age using a cutoff of 50 years.
