# Supplementary material for: Unveiling Silent Atherosclerosis in Type 1 Diabetes: The Role of Glycoprotein and Lipoprotein Lipidomics, and Cardiac Autonomic Neuropathy
Source: Metabolites. 2025 Jan 16;15(1):55. doi: 10.3390/metabo15010055 (PMC11767205; doi:10.3390/metabo15010055)

Supplementary material

Supplementary Figure S1. ROC curves comparing the baseline clinical prediction model and the model incorporating the four significant biochemical metabolites. The baseline clinical model (red line) includes age, duration of diabetes, and smoking status, achieving an AUC of 0.88 (95% CI: 0.84–0.93). The model with the four metabolites (blue line) achieves an AUC of 0.90 (95% CI: 0.86–0.94), although the difference is not statistically significant (DeLong's test, P = 0.1). Performance metrics obtained from 5-fold cross-validation yielded consistent AUC values of 0.88, indicating no substantial improvement with the addition of the metabolites.

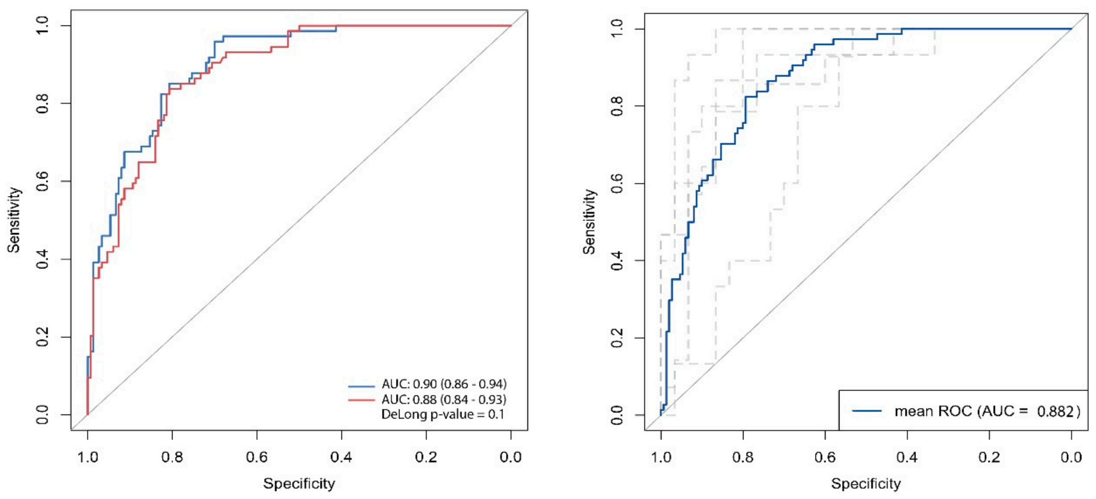

Supplementary Figure S2. ROC curves representing the validation of the predictive models: (A) Baseline clinical model, achieving an AUC of 0.879; (B) Baseline model + GlycA, achieving an AUC of 0.887; (C) Baseline model + HDL-TG, achieving an AUC of 0.885; (D) Baseline model + IDL-TG, achieving an AUC of 0.883; (E) Baseline model + LDL-TG, achieving an AUC of 0.885. Each curve shows the mean ROC across the cross-validation folds, with dotted lines indicating individual fold results.

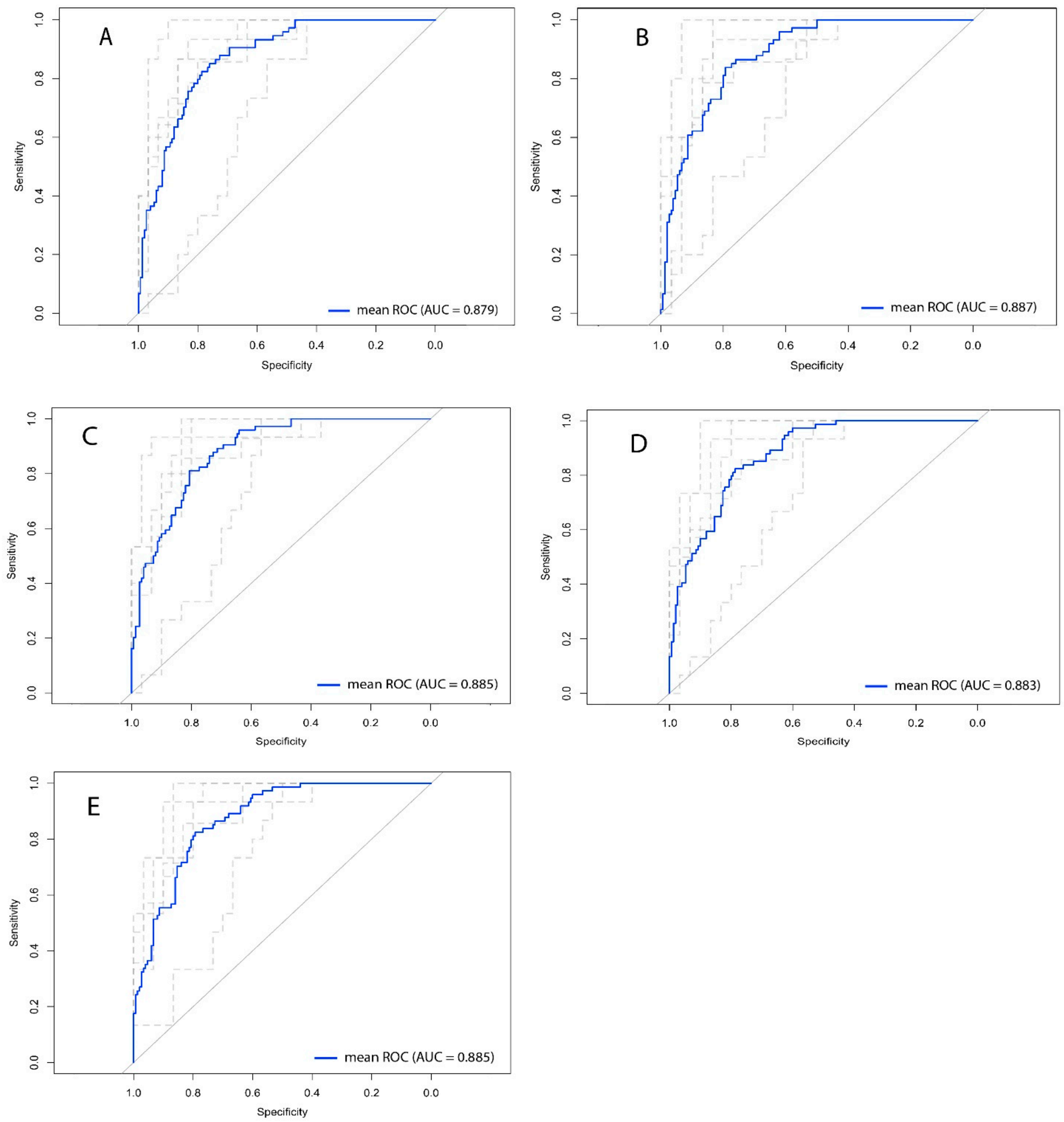

Supplementary Table S1. Confusion matrix for the prediction model at a cut-off point of 0.18. The table summarizes true positives (TP), false positives (FP), false negatives (FN), and true negatives (TN) for plaque prediction. Sensitivity, specificity, positive predictive value (PPV), and negative predictive value (NPV) are reported along with their 95% confidence intervals (CIs), derived from a binomial distribution.

	**Plaque +**	**Plaque -**
**Prediction +**	**69 (TP)**	**55 (FP)**
**Prediction -**	**5 (FN)**	**95 (TN)**

Sensitivity 93% (95%CI 85-98%); Specificity 63% (95% CI 55-71%); PPV 56% (95%CI 46-65%); NPV 95% (95%CI 89-98%)

Supplementary Figure S3. ROC curves comparing the predictive performance of the model adjusted for the presence of cardiac autonomic neuropathy (CAN) [blue line: with CAN; red line: without CAN]. The AUC for the model with CAN is 0.88 (95% CI: 0.81–0.96), and the AUC for the model without CAN is 0.88 (95% CI: 0.83–0.93). The DeLong test for the comparison between the two models yielded a p-value of 0.95, indicating no statistically significant difference in predictive performance based on CAN adjustment.

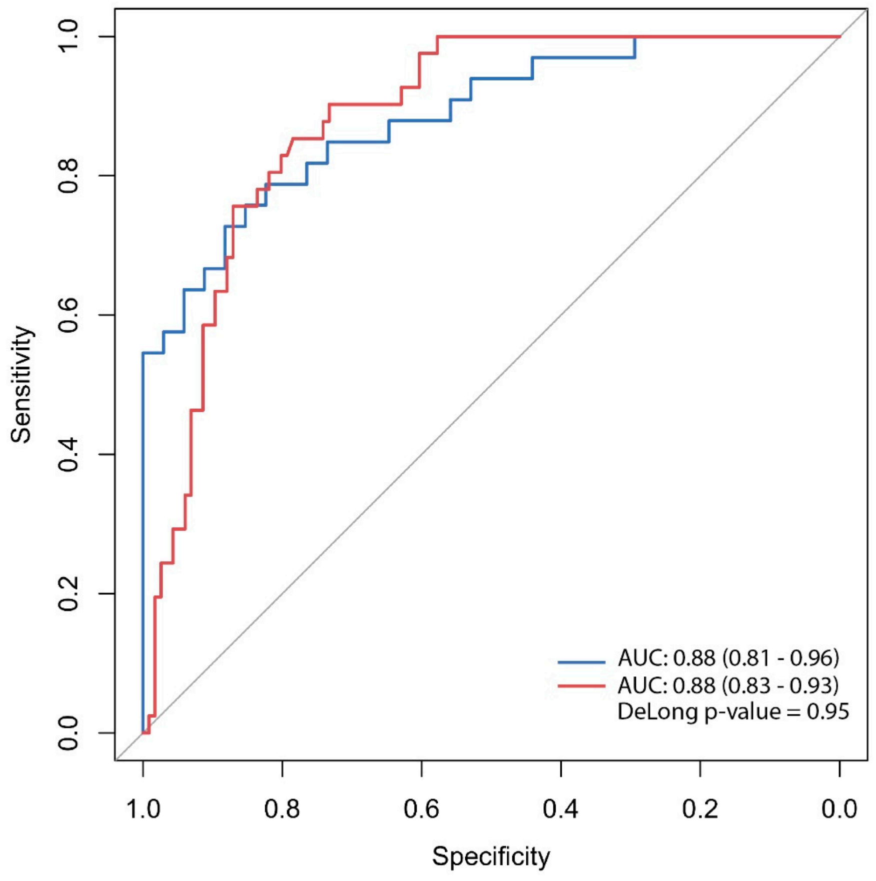

Supplementary Figure S4. ROC curves stratified by age using a cutoff of 50 years. The blue line represents patients under 50 years old (AUC: 0.87, 95% CI: 0.80–0.94), and the red line represents patients aged 50 years or older (AUC: 0.75, 95% CI: 0.64–0.86). The DeLong test p-value of 0.06 suggests a trend toward a difference between the two curves. Smoking history was found to have a greater impact on the model for patients under 50 years of age.

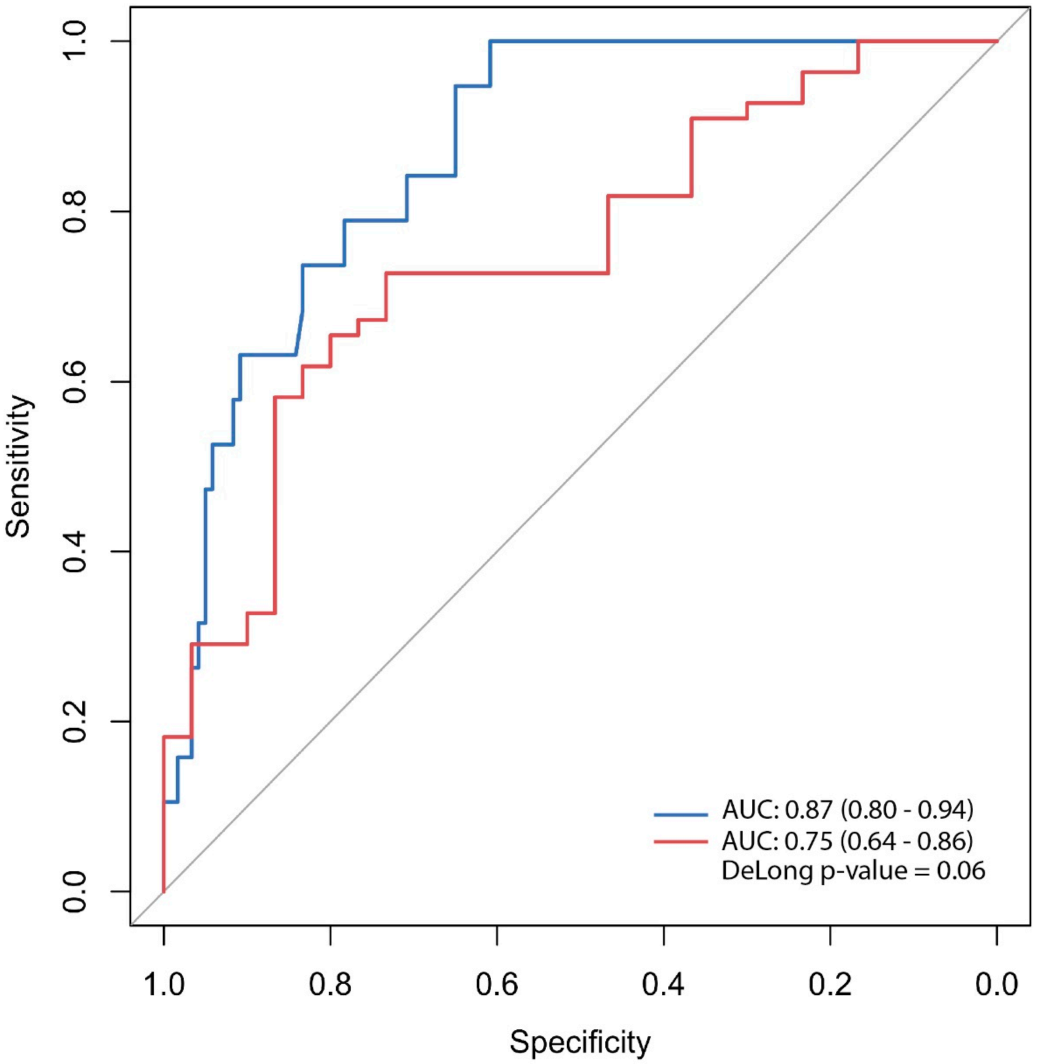

Supplement: Supplementary file 1 [file metabolites-15-00055-s001.zip › metabolites-3424708-supplementary.pdf]
